# Silica Hydrogels as Entrapment Material for Microalgae

**DOI:** 10.3390/polym14071391

**Published:** 2022-03-29

**Authors:** Sarah Vanessa Homburg, Anant V. Patel

**Affiliations:** WG Fermentation and Formulation of Biologicals and Chemicals, Faculty of Engineering and Mathematics, Bielefeld University of Applied Sciences, Interaktion 1, 33619 Bielefeld, Germany; sarah_vanessa.homburg@fh-bielefeld.de

**Keywords:** microalgae, silica, hydrogel, entrapment

## Abstract

Despite being a promising feedstock for food, feed, chemicals, and biofuels, microalgal production processes are still uneconomical due to slow growth rates, costly media, problematic downstreaming processes, and rather low cell densities. Immobilization via entrapment constitutes a promising tool to overcome these drawbacks of microalgal production and enables continuous processes with protection against shear forces and contaminations. In contrast to biopolymer gels, inorganic silica hydrogels are highly transparent and chemically, mechanically, thermally, and biologically stable. Since the first report on entrapment of living cells in silica hydrogels in 1989, efforts were made to increase the biocompatibility by omitting organic solvents during hydrolysis, removing toxic by-products, and replacing detrimental mineral acids or bases for pH adjustment. Furthermore, methods were developed to decrease the stiffness in order to enable proliferation of entrapped cells. This review aims to provide an overview of studied entrapment methods in silica hydrogels, specifically for rather sensitive microalgae.

## 1. Introduction

Microalgae are a diverse group of unicellular organisms including pro- and eukaryotes, freshwater and marine organisms, living individually and in chains or groups. They contain high-value products such as pigments, polyunsaturated fatty acids, vitamins, and polysaccharides, while biodiesel and biohydrogen represent low-value products [1,2,3,4,5,6]. Consequently, they are considered a promising renewable feedstock for the biotechnological production of food, feed, fine and bulk chemicals, and biofuels. Furthermore, they sequester atmospheric CO_2_ and can be produced throughout the year without arable land being required [6,7,8]. Although the first commercialization attempts of microalgal products were undertaken in 1942 [9], the first success was reported in 1957 with *Chlorella* and *Spirulina* as “health food” [10]. However, microalgal production competes with chemical synthesis and biotechnological production with other organisms [1,8]. For this reason, microalgal production focuses on niche markets at the moment. Additionally, limited knowledge about costs on their cultivation and processing at commercial scale is available, and the technology for commercialization is still challenging [4,8]. Consequently, many small- and medium-sized companies disappeared shortly after their foundation [3].

The major obstacle for the commercialization of microalgal products is the high cost of production due to slow growth rates, costly media and photobioreactors, problematic downstream processing, rather low cell densities, and high risk of contaminations [8,11].

A solution is provided by using immobilized microalgae. In this way, cells are effectively separated from the liquid phase, which allows for cultures with high cell densities in comparison with free cells overcoming the disadvantage of slow growth rates. Consequently, harvesting is simplified as well, and the costs of downstream processing are reduced because of the physical separation of the microalgal biomass from the product. Additionally, the separation of biomass and liquid phase enables continuous processes with dilution rates of the bioreactor higher than the microalgal growth rate without the risk of wash-out. Hence, costs for recovery and recycling are reduced as well.

Furthermore, immobilization, especially via entrapment, protects the cells against contaminations with other potentially predatory or competitor microorganisms. In the case of a contamination, the media can be changed easily, preserving the producing microalgae. By analogy, immobilization via entrapment protects the cells against shear forces induced by pumps, valves, or agitators [12,13,14,15,16].

Common applications are the production of metabolites, improvement of culture collections handling, energy production, removal of nutrients or pollutants, and co-immobilization for synergistic effects (for a review see [12]). The application in continuous production processes is especially interesting for secreted products, either by selected wild-type strains or by genetically engineered strains [3,17,18,19].

Immobilization of microorganisms in general has been of interest since approximately 1800, and industrial exploitation has been reported since 1964 [20]. In principle, the methods can be applied to microalgae, taking into account the requirement for light of the photosynthetically active cells and their sensitivity [12,14,15,16]. The immobilization of microalgae was first published in 1966 [21] and has gained more biotechnological interest since approximately 1980 [15,22].

In contrast to adsorption on carriers and aggregation of cells, immobilization via entrapment provides a reduced contamination of the effluent with cells leaking from the carrier. Moreover, covalent or ionic bonds between the cells and to the carrier are omitted, and the cells are protected against contaminations [23,24,25]. For the entrapment, synthetic polymers or biopolymers can be applied in thermal or ionic gelation or complex coacervation [12,13,23,26,27,28].

Entrapment of cells via thermal gelation is limited to cells that tolerate the required temperatures during the gelation process. In general, temperature for gelation should be higher than the temperature for cultivation in order to avoid destabilization of the entrapment matrix during cultivation. As a consequence, if the temperature during cultivation fluctuates too much, e.g., if the temperature is uncontrolled, cells are able to leak out of the destabilized matrix [29,30].

Similarly, entrapment via ionic gelation is limited to cells that tolerate the involved ions in the required concentrations. Furthermore, the entrapment matrix can be destabilized in media with competing ions or by washing out the stabilizing ions in continuous processes [31,32,33].

Entrapment via complex coacervation involves polymers with opposing charges and thus is difficult to control and to predict [25,34,35].

The commonly applied biopolymers or synthetic organic polymers are susceptible to biological destabilization through consumption or degradation by the entrapped cells on the one hand and by contaminants on the other hand. In comparison, inorganic hydrogels derived from silica precursors, such as alkoxysilanes and aqueous silicates, by the sol–gel method are advantageous because of high transparency as well as chemical, mechanical, thermal, and biological stability [36,37].

The sol–gel method involves the formation of a colloidal sol and subsequently an integrated network. Even though the first entrapment of living cells, i.e., *Saccharomyces cerevisiae*, in a silica hydrogel was reported by Carturan et al. in 1989 [38], entrapment of viable sensitive microalgae in biocompatible silica hydrogels remains challenging due to detrimental concentrations of organic solvents, toxic by-products, and mineral acids or bases for pH adjustment [39,40,41]. Furthermore, it has been assumed that proliferation of cells is physically restricted by the stiffness and confines of the silica hydrogel [38,40,41,42,43,44,45,46,47,48,49,50,51], and thus growth of entrapped microalgae is rarely reported [52,53].

While other reviews focus on silica hydrogels and their biocompatibility in general [29,36,37,54,55,56] or on immobilization methods for microalgae [12,15,57], this review aims to provide an overview of studied entrapment methods for rather sensitive microalgae via silica hydrogels. Therefore, first, an overview of the chemical reactions behind the preparation of silica gels is given. In this section, three different routes based on alkoxysilanes, aqueous silicates, and aminosilanes are compared. Second, the consequences for the biocompatibility of the silica hydrogels are pointed out and the reported efforts to increase the biocompatibility are described. Third, the applied methods for microalgae entrapment are summarized.

## 2. Sol–Gel Methods for the Production of Silica Hydrogels

### 2.1. Sol Synthesis with Alkoxysilanes

Conventional precursors for sol–gel methods are alkoxysilanes such as tetraethyl orthosilicate (TEOS) or tetramethyl orthosilicate (TMOS). In contrast to aqueous silicates (see the next paragraph), the condensation occurs while hydrolysis has not yet been completed.

Under acidic conditions, hydrolysis starts with protonation of the alkoxy group, making it easier for water to attack the silicon and the alcohol demerged by a nucleophilic substitution. The tetrad structure of the precursor is inverted in the meantime (see Equation (1)).
(1)
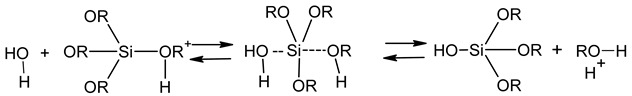


At the same time, condensation starts with a protonation of the silanol group according to Equation (2).
(2)
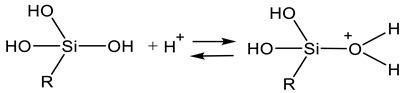


The resulting silanolate-cation reacts with another silanolate group to a siloxane bond through separation of an oxonium ion according to Equation (3).
(3)
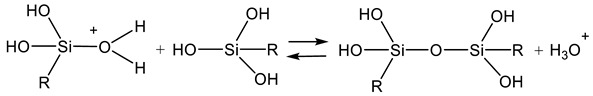


As the hydrolysis degree increases, the hydrolysis and the condensation rate decrease [58]. For this reason, monomers and terminal groups are preferable in hydrolyzed form [59,60]. In total, the hydrolysis rate is greater than the condensation rate. As a result, long, hardly branched chains are generated, which grow to 2–3 nm before a network is built and gelation starts [60].

Under alkaline conditions, a hydroxide ion attacks the silicon via a nucleophilic substitution. Subsequently, an alkoxide ion separates on the opposite site. Analogous to the acid catalysis, an inversion of the silicon’s tetrad structure occurs (see Equation (4)).
(4)
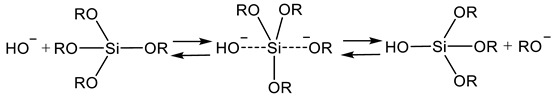


The condensation at alkaline conditions starts with the deprotonation of the silanol group via separation of water (Equation (5)).
(5)
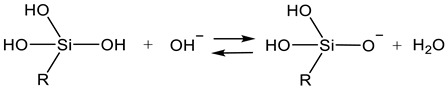


The resulting silanolate anion reacts with another silanol group to a siloxane bond, analogous to acid conditions. Likewise, the reaction is supposed to follow an S_N_2 mechanism (Equation (6)).
(6)
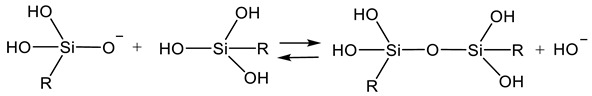


**Figure 1:**
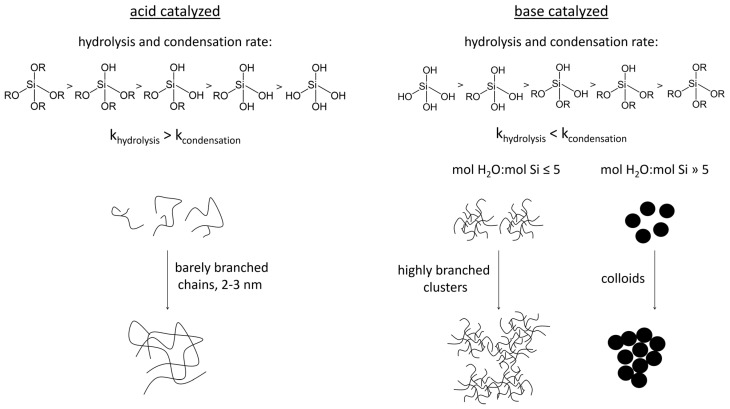
Schematic presentation of hydrolysis and condensation of alkoxysilanes under acidic and alkaline conditions, modified from [58,60,61,63].

In contrast to the acid catalysis, at alkaline conditions, the condensation rate is higher than the hydrolysis rate. Furthermore, both rates increase with an elevated degree of hydrolysis, and therefore highly branched clusters develop [59,60,61]. At alkaline conditions and high molar water to silica ratios, colloids develop that grow via Ostwald ripening until they are stabilized by their surface loading (Stöber process) [58]. The size of the colloids depends on the solvent and the ratio of solvent to water [62]. The impact of the conditions on the structure is schematically displayed in Figure 1.

### 2.2. Sol Synthesis with Aqueous Silicates

**Figure 2:**
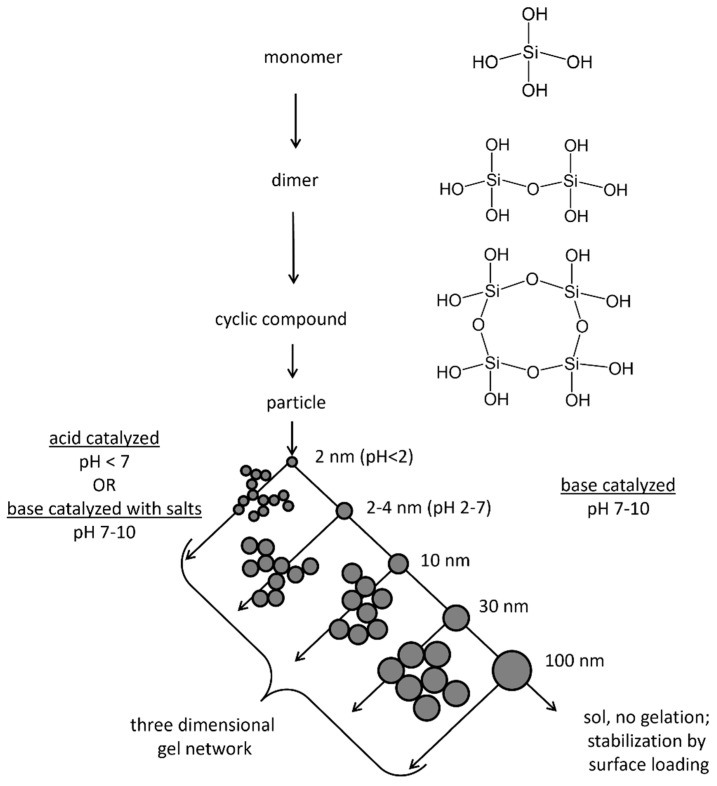
Schematic presentation of the condensation of aqueous silicates, modified from [55,60].

Aqueous silicates such as sodium silicate belong to the conventional precursor of the sol–gel method and are therefore well known [58,61,63,64,65]. After dilution of the precursor in water, monomers of silicic acid develop that rapidly condense. The monomers polymerize to particles by maximization of the Si-O-Si bonds and minimization of terminal hydroxyl groups. For this reason, a ring formation initially occurs, to which further monomers are added, resulting in a three-dimensional particle. Subsequently, the particles grow by Ostwald ripening. The particles’ size depends on the pH and the presence of salts [58]. This is schematically displayed in Figure 2.

At acidic pH, the solubility of a particle is low, and therefore the particles grow up to 2–4 nm before they are connected first to chains and afterward to networks. These networks spread in the aqueous medium before they finally gel. If the pH is lower than 2, formation and aggregation of particles occur at the same time. After the particles have reached a size of 2 nm, the Ostwald ripening stops, and a network is formed from these small particles.

In contrast, at alkaline conditions, the solubility of the particles is higher. Furthermore, the condensed particles are charged, and thus they repel each other. For this reason, an enhanced growth via Ostwald ripening instead of a connection of the particles occurs. In the absence of salts, aggregation is lacking and a stabilized sol is developed [58,64].

At a pH greater than 2, condensation starts with the deprotonation of the silanol group by a hydroxide ion (Equation (7)). Already condensed species are more likely ionized.
(7)
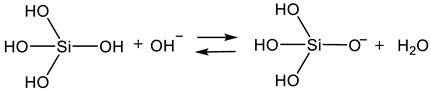


Afterward, the silanolate anion reacts with another silanol group via separation of a hydroxide ion (Equation (8)). In comparison, the first reaction (Equation (7)) occurs faster than the second (Equation (8)) [58].
(8)
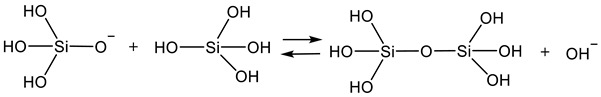


During acid catalysis, growth according to Equation (8) occurs preferably with highly condensed and less condensed species, while during base catalysis, the charged condensed species repel each other. This is why at a pH greater than 7, the addition of monomers is favored [58].

During the condensation at a pH smaller than 2, the addition of a proton leads to a partially positively charged intermediate according to Equation (9).
(9)
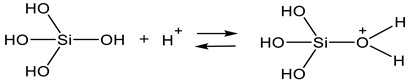


The intermediate reacts with another silanol group via separation of an oxonium ion (Equation (10)).
(10)
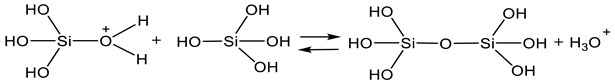


### 2.3. Sol Synthesis with Aminosilanes

In the case of the precursor tetra(*n*-propylamino)silane as a representative of tetra(alkoxyamino)silanes, investigations on the ammonolysis and the subsequent condensation to NSi_3_ and HNSi_2_ networks are reported [66,67]. It was observed that the precursor as well as the occurring by-product *n*-propylamine function as a base and therefore cause the autocatalysis of the precursor [68]. Furthermore, Si-N bonds show a smaller bond energy of 437.1 ± 9.9 kJ/mol in comparison to Si-O bonds of 799.6 ± 13.4 kJ/mol [69]. For this reason, the reactivity of tetra(*n*-propylamino)silane is greater than that of alkoxysilanes such as TEOS [70]. As a consequence of the autocatalysis and the smaller bond energy, the precursor reacts to the addition of water with a rapidly formed white precipitation that was identified as (Si_x_O_y_)_z_ [70]. This reaction (Equation (11)) takes place at the interface of the tetra(*n*-propylamino)silane emulsion droplets.
(11)
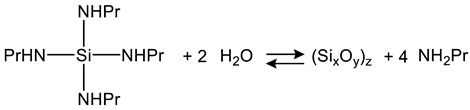


The developed (Si_x_O_y_)_z_ agglomerates and the emulsion droplets of the precursor form a turbid dispersion in the aqueous medium. The increasing alkalinity based on the developed by-product causes a fragmentation of the agglomerates according to Equation (12) and dilutes in the aqueous solution. The resulting polydisperic sol is transparent and displays particles comparable with basic catalyzed TEOS gels [70].
(12)
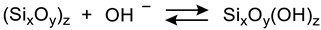


**Figure 3:**
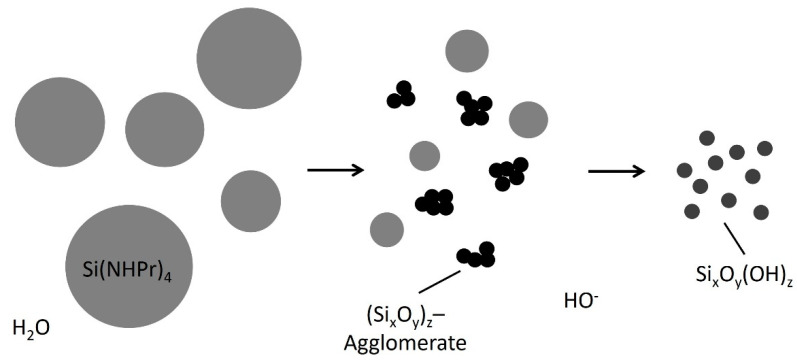
Schematic representation of the reaction of tetra(*n*-propylamino)silane with water, modified from [68,70].

The described development of a particulate sol in aqueous media at basic conditions is displayed in Figure 3.

### 2.4. Gel Synthesis

Entrapped cells are affected by the gel time due to sedimentation of the cells and delay until the hydrogel can be covered with cultivation medium to supply nutrients [41]. The gel time is specified as the time until the gel point is reached, which in turn is defined as the time when an infinite, spanning polymer or aggregate first appears [71]. Consequently, the gel time is a function of the hydrolysis and condensation rate and therefore depends on the pH value as well [58,72]. In case of alkoxysilanes, the rate of hydrolysis is linearly proportional to the concentration of acid and base [72]. Below the isoelectric point, i.e., pH 2, the rate of hydrolysis is large compared to the rate of condensation. Consequently, at large molar water/silica ratios (H_2_O:Si > 4), the hydrolysis will be complete at an early stage of the reaction [58]. At intermediate pH conditions, i.e., between pH 3 and pH 8, the rate of condensation increases with the pH value, while the rate of hydrolysis goes through a minimum at approximately neutral pH. As a consequence, hydrolysis is rate-limiting for gelation at these pH conditions [58,72]. Above pH 7, condensation occurs by nucleophilic displacement reactions via SiO^−^ anions, preferentially between protonated and deprotonated acidic species [58]. For this reason, the rate of condensation decreases with increased pH due to mutual repulsion.

**Figure 4:**
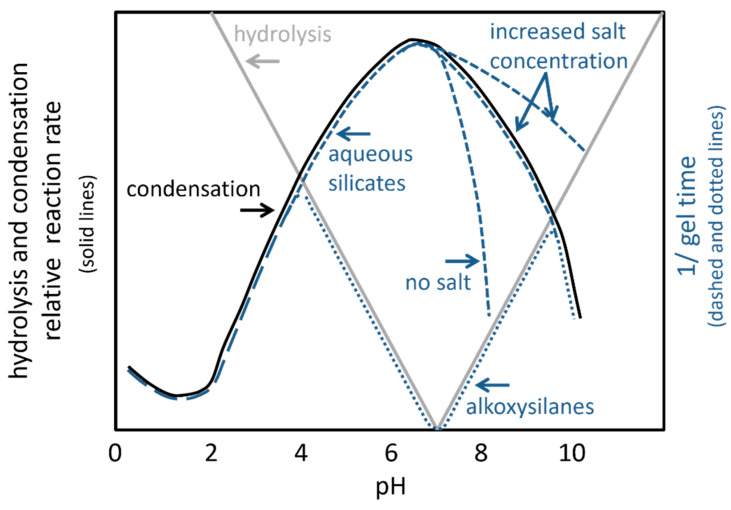
Schematic representation of hydrolysis and condensation rate as well as gel time in dependence of the pH; from [58,64,72,73].

In the case of diluted aqueous silicates, hydrolysis is immediately and fully completed at all pH conditions. Hence, the gel time solely depends on the condensation time. Around pH 1.5–3, sols display a maximum stability, and therefore the longest gel time. The rate of condensation is proportional to the concentration of protons. Between pH 2 and 7, the condensation rate is proportional to the concentration of hydroxide ions. Consequently, the gel time decreases with the pH, with a minimum at pH 5–6. Above pH 7, the particles are charged and therefore mutually repulsive. For this reason, the particles grow, but no gelation can be observed. The addition of salts lowers the ionic charge on particles, and therefore a gelation is possible and the gel time decreases [58,64]. The relative reaction rates of hydrolysis and condensation as well as the gel time are displayed in Figure 4.

**Figure 5:**
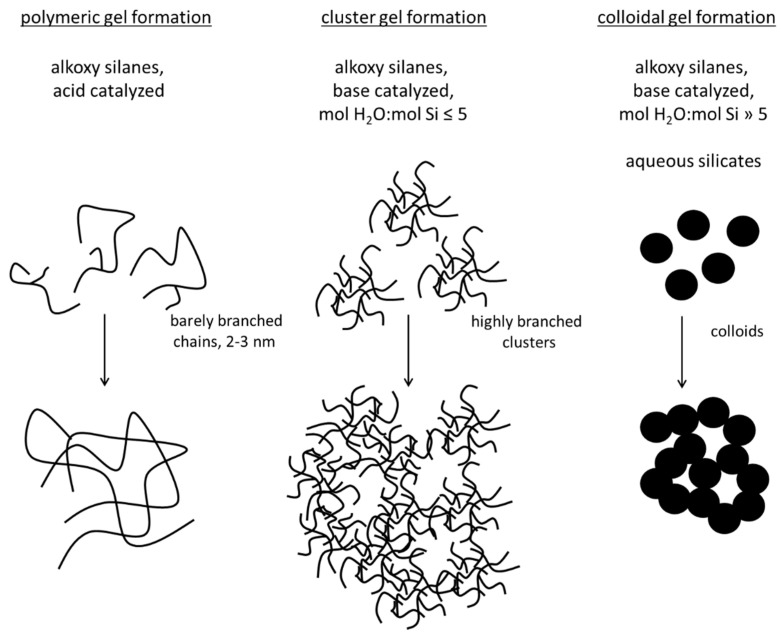
Schematic presentation of the polymeric, cluster, and colloidal gel formation, modified from [60,61,64].

Besides the gel time—the time until nutrients can be added to the entrapped cells—the diffusion rate of nutrients and therefore the gel structure affects survival, growth, and productivity of entrapped cells. The gel structure, more precisely the pore size and its distribution, depends on the gel formation process [61]. Three gel formation processes are mainly distinguished, i.e., polymeric, cluster, and colloidal gel formation (Figure 5).

Polymeric gel formation occurs with alkoxysilanes as precursor at low molar water to silica ratios (H_2_O: Si ≤ 5) and acid catalyzed conditions. The linear or randomly branched polymers entangle and form additional branches resulting in gelation.

In contrast, at high molar water to silica ratios and/or base catalyzed conditions, the highly branched clusters do not interpenetrate before gelation and thus behave like discrete species. Gelation occurs by linking together clusters similar to colloidal gel formation [61].

Colloidal gel formation is a commonly known process based on aqueous precursors. As briefly described in the previous chapter, gelation of aqueous colloidal particles occurs only at pH values below 7 or in the presence of salts. When two particles collide, neutral and protonated silanol groups on the surface of the particles condense to form Si-O-Si linkages. In the presence of soluble silica or monomers, the particles are cemented together [64].

## 3. Biocompatibility

The biocompatibility of silica hydrogels is limited for three main reasons:

First, alkoxysilane precursors such as TMOS or TEOS are poorly soluble in water, and therefore the conventional sol synthesis applies organic solvents, e.g., the alcohol that is already released during the hydrolysis.

Second, osmotic stress is generated by the addition of acids or bases as catalysts to increase the reaction speed during hydrolysis and condensation and to modify the molecular structure of the resulting hydrogel. Further ions are added by adjusting the pH of the sol and therefore of the resulting hydrogel with mineral bases or acids.

Third, the stiffness of conventional silica hydrogels is discussed to limit or even prevent proliferation [38,40,41,42,43,44,45,46,47,48,49,50,51,74,75,76].

### 3.1. Conventional Sol–Gel Method for the Entrapment of Insensitive Biological Material

For these reasons, the entrapment by the conventional sol–gel method is limited to biological material that tolerates the applied solvents, the released by-products, and the applied acids or bases for catalysis and pH adjustment. The first pioneering studies reported the entrapment of *Saccharomyces cerevisiae* by Carturan et al. in 1989 [38] and of the alkaline phosphatase by Braun et al. in 1990 [77]. In both cases, the entrapped biological material tolerates the applied and the released alcohol. Hence, the entrapped enzyme as well as the yeast cells were reported to show activity; however, this was reduced to free biocatalysts. This is why the synthesis conditions were assumed to be in principle biocompatible by these authors.

The biocompatibility of silica hydrogels was increased by omitting alcohol as a solvent on the basis of the observation that the alcohol released during hydrolysis sufficiently increased the solubility of the precursor [78].

### 3.2. Reduction of Released By-Products and Avoidance of Increased Ion Concentrations

**Figure 6:**
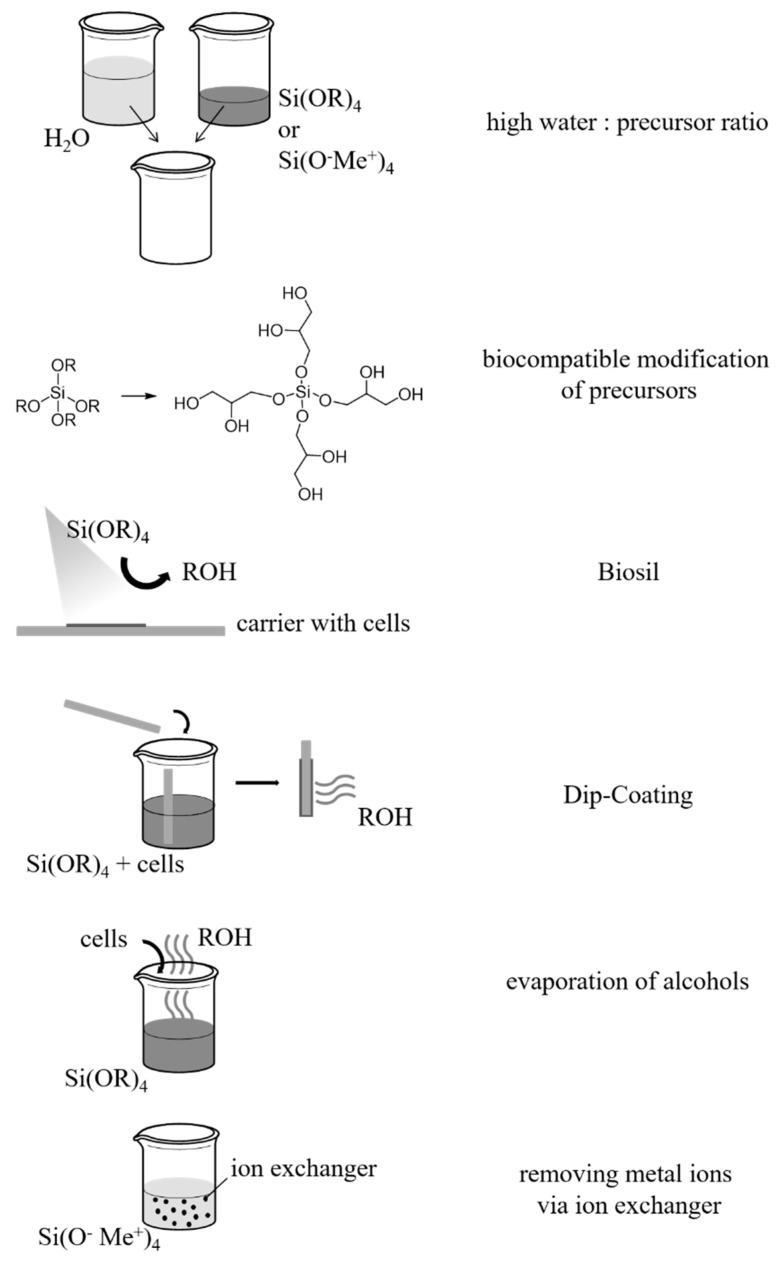
Schematic representation of methods for mitigating the concentration of by-products in order to increase the biocompatibility of the sol–gel method for hydrogel production.

As reviewed by Coradin and Livage [39], the limitation of biocompatibility due to the release of the alcohol as a by-product during hydrolysis can be reduced by different methods (see Figure 6):

- mixing water and precursor in a high ratio [79];
- modification of the precursor with biocompatible alkoxides, e.g., to poly(glyceryl silicate) [80];
- application of the precursor from the gas phase during which the alcohol evaporates and contact with the entrapped material is avoided (Biosil method) [54,55,81,82,83,84];
- dip-coating of a carrier in order to create a thin layer of a few micrometers from which the released alcohol can evaporate quickly from the close proximity of the entrapped biological material [38,85,86];
- evaporation of the alcohol released from the sol before the biological material is added [44,55,76,87,88,89,90,91].

Another method to avoid organic solvents is the application of aqueous silicates as precursors for synthesis. On the one hand, the disadvantages compared to alkoxysilanes originate in the limitation of the precursor concentration and the pH as well as in the reactions that are difficult to control. The latter is caused by the mixture of oligomers, while alkoxysilanes exist in the solution as monomers. On the other hand, the advantage of aqueous silicates originates in the metal ions that are released as by-products. Since they occur naturally, some microorganisms show a tolerance towards them [39]. For the entrapment of cells that are sensitive to the induced osmotic stress, the cations are removed via strongly acidic ion exchangers [43,44,76,92,93,94,95,96] (see Figure 6).

Another possibility to avoid organic solvents as well as acids and bases as catalysts arose from the novel silica precursor tetra(*n*-propylamino)silane, which exhibits higher reactivity when compared to the use of alkoxysilanes [70].

In order to adjust the pH of the sol and hence of the silica hydrogels, mineral acids and bases, e.g., hydrochloride acid, potassium, sodium, or ammonium hydroxide are conventionally applied [41,42,46,51,97,98,99,100,101,102,103]. Alternatively, the pH is adjusted by resolving the biological material in specific buffers and mixing the solution in an appropriate amount of the sol so that the desired pH is reached [36,55,89,92,93,99,104,105,106,107,108].

### 3.3. Facilitation of Cell Proliferation by Reducing the Stiffness

In the past years, yeasts such as *S. cerevisiae* [38,45,86,102,109,110,111], bacteria such as *Escherichia coli* and *Bacillus subtilis* [40,47,48,51,85,88,93,99,100,101,103,104,107,108,112,113,114,115,116,117,118,119,120,121,122,123], cyanobacteria such as *Synechococcus* or *Synechocystis* [41,46,76,94,97], and microalgae such as *Chlorella vulgaris* [42,43,44,74,75,98,124,125,126,127,128,129,130,131,132,133] have been entrapped in silica hydrogels. Despite the numerous publications on the entrapment of whole cells in silica hydrogels, proliferation and growth has been barely reported [85,86,103,109,116]. On the one hand, on the basis of the application as a biosensor, cell growth has not been a focus of research. On the other hand, a commonly known disadvantage of silica hydrogels is their stiffness [80,134,135,136,137,138], which is discussed to limit or even prevent proliferation [38,40,41,42,43,44,45,46,47,48,49,50,51,74,75,76].

In order to enable cell growth, a two-step method has been developed. Here, cells were entrapped in a biopolymer hydrogel that was afterward entrapped in silica hydrogels [111,117,119,126,139].

Alternatively, composites (also called hybrids) of silica hydrogels and biopolymers have already been investigated. Furthermore, organic or biological additives increase the stability and bioactivity of the entrapped biological material [95], for example by creating a hydrophilic environment [138]. As described by Coradin et al. [56,89], the most frequently added polymers are proteins such as gelatin [55,91,100,101,140,141,142,143,144,145] and polysaccharides such as cellulose [50,55,136,143], alginate [39,49,54,55,89,95,99,143,144,145], and chitosan [55,108,136,137,138,142,143,146,147,148,149,150,151,152,153,154,155,156,157,158,159,160,161,162].

Besides biopolymers, the synthetic polymer polydiallyldimethylammonium chloride (PDADMAC) was utilized for the production of composites: cells were entrapped in an alginate–silica composite by dropping a mixture of cells, sodium alginate, and a silica precursor into a mixture of calcium chloride and PDADMAC. As a result, a double network of calcium alginate and silica hydrogel with a shell of PDADMAC was created [96,131,132,133], in which cell growth was enabled [131].

## 4. Entrapment of Microalgae in Silica Hydrogels

### 4.1. Entrapment of Microalgae in Alkoxysilanes

Alkoxysilanes, mostly TEOS and rarely TMOS, have been applied for the entrapment of the green microalgae *Chlorella vulgaris*, *Haematococcus pluvialis*, and *Chlamydomonas reinhardtii*, as well as the cyanobacteria *Anabaena* and *Synechocystis* sp. Applied TEOS concentrations of 5 wt % to 22 wt % released ethanol in concentrations of 0.92 to 4.24 mol/L, while applied TMOS concentration of 6.87–11.70 wt % released 4.6–7.88 mol/L methanol. In order to reduce the detrimental effect of the released alcohols, dip-coating of a carrier to create a thin layer [75] as well as evaporation of alcohols were applied [46,52,53,74,76]. In all reviewed studies, sol–gel synthesis was acid catalyzed, resulting in barely branched chains and a polymeric gel formation (see Section 2.1 and Section 2.4). Where necessary for increased biocompatibility and for induction of gelation, the pH was adjusted with NaOH, KOH, or phosphate buffer (see Table 1). In one study, a polyether-modified polysiloxane enhanced the mechanical stability, which led to decreased cell loss of the thin layer [75].

Furthermore, organically modified silanes, so-called ORMOSILs, were employed. In this way, the presence of a covalently bound functional group can alter the structure of the matrix by reducing subsequent cross-linking as well as hydrolysis and condensation reactions through electrostatic interactions and steric hindrance. These modifications were reported to increase the mechanical stability [74,75]. Additionally, the functional group can alter the interaction between the hydrogel and the pore fluid and encapsulated cells. For example, MTES induced hydrophobic methyl groups into a generally hydrophilic matrix. Interactions between the resulting hydrogel and entrapped cells depended on the cells’ hydrophobicity [46]. Consequently, viability and cell growth were limited and species-specific. While *Chlorella vulgaris* and *Anabaena* sp. cells entrapped in MAPTS (3-(trimethoxysilyl)propyl methacrylate)/TMOS or MTMOS (methyltrimethoxysilane)/TMOS/PhTMOS (phenyltrimethoxysilane) gels showed signs of cell death [74], *Haematococcus pluvialis* cells entrapped in TEOS/GLYEO ((3-glycidyl-oxypropyl)triethoxysilane) gels were viable for 40 days and able to produce the carotenoid dye astaxanthin [75].

In order to further increase the biocompatibility, glycerol, sorbitol, and/or polyethylene glycol were added to the gels. Addition of sorbitol or glycerol stabilized cell vitality of *Haematococcus pluvialis*, also upon astaxanthin extraction with solvents [75]. While glycerol had a negligible effect on hydrogen production in wild-type *Synechocystis* sp. cells and hindered hydrogen production in the mutant cells, polyethylene glycol 400 improved hydrogen production. Finally, hydrogen production was observed for 5 days, similar to free cells [46].

In comparison with silica hydrogels prepared via other routes, photosynthetic activity of *Synechocystis* sp. was diminished and less stable in TEOS-derived silica hydrogels [46,76]. Similar observations were reported for the microalga *Chlamydomonas reinhardtii* [52,53].

Besides additives, one study investigated the application of the alkoxide precursor tetrakis(2-hydroxyethyl)orthosilicate with the more biocompatible by-product glycolic acid. The precursor was applied in a concentration of 12 wt % to immobilize the microalga *Porphyridium purpureum*. For the sol synthesis, no additional acid or base was necessary. The gelation occurred upon mixing with the cells at pH 6. The entrapped cells showed a stable viability at elevated temperatures as well as pigment fluorescence over two weeks. Hence, the potential application as whole-cell biosensor for aqueous contaminants was demonstrated [163].

### 4.2. Entrapment of Microalgae in Aqueous Silicates

A possibility to avoid the inhibitory by-products of alkoxysilanes is the application of aqueous silicates such as potassium, lithium, or sodium silicates. Here, the metal ions occur as by-products. Commonly applied sodium silicate concentrations of 1.06–19 wt % resulted in sodium ion concentrations of 0.64 to 18.8 mol/L [41,42,97,98,124,129,130,164,165]. It has already been applied for the entrapment of the microalgae *Chlorella vulgaris*, *Dictosphaerium chlerelloides*, *Scenedesmus intermedius*, *Scenedesmus* sp., *Mesotaenium* sp., and *Cyanidium caldarium* as well as the euglenoid *Euglena gracilis* and the cyanobacteria *Anabaena flos-aqua*, *Synechococcus* sp., and *Cyanothece* [41,97,98,124,129,130].

In all reviewed studies (see Table 2), the sol synthesis was base catalyzed, and the pH was adjusted with HCl, resulting in colloidal gel synthesis (see Section 2.2 and Section 2.4). In most of the studies, the colloidal suspension of nanoparticulate silica, i.e., LUDOX^®^, was added to the sol in order to increase the silica content and reinforce the gel. However, one study indicates that the colloidal silica adsorb on the cell wall, forming a crust that potentially blocks active transport sites and limits cell activity of *C. caldarium* cells. Moreover, the authors argued that the nanoparticles are small enough to be potentially internalized [42]. This effect seems to be species-specific, as other microalgae and cyanobacteria show cell activity in LUDOX^®^-reinforced silica hydrogels [97,98,124,129,130,164,165]. It was discussed that observed harmful effects of elevated LUDOX^®^ concentrations from 1 to 3 mol/L on entrapped *Anabaena flos-aquae* could be caused by an increased Young’s modulus [130].

In order to prevent osmotic shock induced by the upcoming sodium ions, glycerol was added to the sol or to the cells before mixing with the sol in some studies [41,42,97,98,124]. Furthermore, minimizing cracks in the gel microstructure and improving mechanical properties have been discussed [98,124]. However, while in some cases glycerol slows down the rate of degradation of the photosynthetic pigments within the cyanobacteria cells and consequently preserves the viability [41], in other cases, no effect of glycerol on chlorophyll fluorescence was observed [98,124], and for some organisms and strains, the addition of glycerol is not even biocompatible [42,97]. The observed detrimental effect of glycerol was attributed to reduced surface area and pore volume, hence closing pores that potentially reduce diffusion of nutrients [41].

The reviewed studies aimed at whole-cell biosensors for aqueous contaminants on the basis of the fluorescent properties of photosynthetic pigments or presented the results as a first step toward important biotechnological applications, such as biofuels and (secondary) metabolites, however, without giving insights on specific products. All studies reported the viability of cells, while a proliferation was only observed for *Synechococcus*. However, proliferation was limited to two generations, which the authors attributed to space limitations in the silica hydrogel [41,97].

### 4.3. Entrapment of Microalgae in Aqueous Silicates with Metal Ion Removal

In order to further increase biocompatibility, sodium ions can be removed with an ion exchanger before the cells are added to the sol, resulting in the so-called “low-sodium route.” Consequently, higher precursor concentrations, i.e., 4.8–25 wt %, were applied in comparison to 1.06–19 wt % without ion removal (see the previous paragraph). By applying an ion exchanger, the sol acidifies, causing acid catalysis.

However, when LUDOX^®^ is added to the sol in order to strengthen the gel analogous to the previously described sodium silicate-based gels, the sol turns alkaline again. Therefore, the pH was adjusted with HCl. With this method, two strains of the cyanobacterium *Synechococcus* sp. have been entrapped. While the addition of the nanoparticles has a beneficial effect on the viability of the cells over time, the mechanical stability was enhanced upon depletion of LUDOX^®^. In fact, the gel prepared with the medium low in sodium ions for the freshwater *Synechococcus* strain was reported to liquefy more quickly than the gel prepared with the salt-water medium for the marine strain. This indicates the need of sodium ions for gel formation in presence of silica nanoparticles.

Since the sodium ions of the silicate precursor were removed via an ion exchanger and LUDOX^®^ was omitted, glycerol was no longer necessary to prevent osmotic stress of the cells. With this method, entrapped cyanobacteria cells produced oxygen for 17 weeks [94]. Similarly, the addition of glycerol and polyethylene glycol was observed to be detrimental toward entrapped *Synechocystis* sp. cells. Nevertheless, cells entrapped in silica hydrogels prepared via the “low-sodium route” displayed a higher vitality than entrapped in TEOS-derived hydrogels. Furthermore, photosynthetic activity was reported for 8 weeks in aqueous silica gels compared to 6 weeks in TEOS-based hydrogels [76].

As an alternative to LUDOX^®^, SiO_2_ nanopowder was added to the sol to strengthen the gel. The pH of the sol remained acidic upon addition of the nanopowder, and thus the pH was adjusted with KOH. In contrast to LUDOX^®^, the aggregates of the nanopowder were too large to be internalized within the cell and were shown to maintain the amount of viable *Cyanidium caldarium*, *Chlorella vulgaris*, and *Botryococcus braunii* cells and their activity in the hydrogel. Furthermore, the nanopowder not only delayed liquefaction of the gels, but also increased diffusion. It was discussed to be caused by the created void pockets around the silica aggregates found in close proximity to the cells. Microalgae entrapped via this method showed oxygen production for 75 days [43,44].

Despite the improvement of the viability by removing the sodium ions, cell growth was still limited, which was again attributed to space limitations [43]. The application of the low-sodium route together with the addition of chitosan as well as pH adjustment with tris(hydroxymethyl)aminomethane allowed entrapment of viable and growing *Chlamydomonas reinhardtii* [52,53].

In all reviewed studies, the authors aimed at enabling viability and activity for important biotechnological applications, such as biofuels and (secondary) metabolites with simultaneous CO_2_ mitigation (see Table 3).

### 4.4. Entrapment of Microalgae in Aminosilane-Based Silica Hydrogels

The precursor tetra(*n*-propyl amino)silane, an aminosilane precursor, is reported to autocatalyse. The emerged sol displays particles comparable with basic catalyzed TEOS gels (see Section 2.3).

This precursor has already been employed for the entrapment of *C. reinhardtii* without morphological changes of entrapped cells. However, the quantum yield of photosystem II and the oxygen consumption rate were drastically reduced, and an oxygen production was not observed. The investigation stopped observation after 2 h after entrapment [70]. The by-product of this precursor, i.e., *n*-propylamine, can act analogously to the herbicide atrazine [166,167,168,169]. Analogous to the low-sodium route for aqueous silicates, *n*-propylamine can be removed via ion exchanger. Hydrogels prepared via this low-propylamine route enabled the entrapment of photosynthetically active and growing *Chlamydomonas reinhardtii* cells (see Table 4) [52,53].

### 4.5. Core-Shell and Two-Step Entrapment of Microalgae

In order to overcome the stiffness of the silica hydrogels that are discussed to limit cell proliferation of entrapped cells, hybrid core-shell beads were employed for the entrapment of the microalgae *Dunaliella tertiolecta* and *Chlamydomonas reinhardtii*. Therefore, an aqueous sol was cleared of sodium ions with the help of an ion exchanger and pH adjusted to 5.1 with NaOH, mixed with sodium alginate and the cells, and finally dropped into a mixture of PDADMAC and calcium chloride to induce condensation of the silica precursor and gelation of the alginate. Moreover, PDADMAC builds an external layer by ionic polymer coating around the beads that enclosed the rigid silica hydrogel. Entrapped cells were observed to produce oxygen for 4 and 13 months in cases of *C. reinhardtii* and *D. tertiolecta*, respectively. However, no cell growth was reported [96,132,133].

Viability of entrapped cells for several weeks or even months as well as c growth of entrapped green microalgae has been reported when *Chlorella vulgaris*, *Pseudokirchneriella subcapitata*, and *Chlamydomonas reinhardtii* were entrapped via a two-step method: in the first step, the cells were entrapped in calcium alginate beads, which were entrapped in silica hydrogels in a second step. As silica precursor for the second step, either TEOS with the evaporation of released alcohol [126,127], in one case in combination with a diamine-functionalized silane [127], or sodium silicate with the addition of LUDOX^®^ was applied [125,128,170]. Mostly, the authors envisioned the application of the entrapped cells as whole-cell biosensor for aqueous contaminants (see Table 5).

## 5. Conclusions

Despite being a promising entrapment material due to the high transparency and biological, chemical, mechanical, and thermal stability, silica hydrogels still display a limited biocompatibility caused by the by-products that are released during hydrolysis of the precursors, by pH adjustment of the sol with mineral bases and by the stiffness of the corresponding hydrogel.

As a closer look at microalgae entrapment in the previous chapter reveals, the development of biocompatible silica hydrogels focused on the precursor selection, the choice of acid or base for pH adjustment, and the addition of other (bio)polymers. While pigment stability, photosynthetic activity, bioactivity, and cell growth have been investigated, the transparency and stiffness of the hydrogel have rarely been reported.

On the basis of the given overview, an in-depth understanding of how the sol and hydrogel synthesis as well as the resulting structures affect the viability, activity, and proliferation capability of entrapped microalgae cells is still needed.

Future developments of biocompatible silica hydrogels for the entrapment of sensitive, photosynthetically active microorganisms may include the synthesis of novel silica precursors that release non-toxic by-products or by-products that even improve viability and/or growth of the entrapped cells. Further organic and inorganic compounds may be screened for their potential application as plasticizers of silica hydrogels in order to decrease stiffness, improve abrasion resistance, and increase the growth capacity of entrapped cells.

## Figures and Tables

**Table 1:** Overview of microalgae entrapment in alkoxysilanes. TEOS: tetraethyl orthosilicate; GLYEO: (3-glycidyl-oxypropyl)triethoxysilane; MTES: methyltriethoxysilane; MAPTS: 3-(trimethoxysilyl)propyl methacrylate; TMOS: tetramethyl orthosilicate; MTMOS: methyltrimethoxysilane; PhTMOS: phenyltrimethoxysilane.

Silica Precursor and Concentration	Catalysis	pH Adjustment	Additives	Microalgae/ Cyanobacteria	Characteristics/ Viability	Purpose/Aim	Ref.
TEOS: 0.45 mol/L/2.27 wt % or 1.79 mol/L/9.25 wt % TEOS/GLYEO: 0.4 mol/L/2.05 wt %	acid catalysis	--	glycerol, sorbitol, polyether-modified poly-siloxane	*Haematococcus pluvialis*	entrapped cells viable for more than 40 days	continuous production of the carotinoid dye astaxanthin	[75]
TEOS: 1.06–1.70 mol/L/6.46–15.55 wt % TMOS: 1.15–1.97 mol/L/6.87–11.70 wt % MTES: 1.09–1.79 mol/L/6.67–11.14 wt %	acid catalysis with HCl or HNO_3_	adjusted to 8 with NaOH or KOH	glycerol or PEG 400	*Synechocystis* sp. PCC 6803 wild-type and mutant M55	H_2_ production for 5 days similar to free cells	enabling (prolonged) viability and activity for important biotechnological applications, such as biofuels and (secondary) metabolites, here H_2_	[46]
TEOS: 1.70 mol/L/10.56 wt %	acid catalysis	adjusted by high cell to sol ratio	glycerol or PEG 200	*Synechocystis* sp. PCC 6803	viability, photosynthetic activity over 6 weeks		[76]
MAPTS/TMOS: 2.75 mol/L/17.38 wt %; MTMOS/TMOS/PhTMOS: 2.51 mol/L/15.23 wt %	acid catalysis	--	--	*Chlorella* *vulgaris*, *Anabaena* sp. PCC7120	no viability upon entrapment	electrochemical sensors; bioremediation with non-living tissue	[74]
TEOS: 0.23–1.06 mol/L/5–22 wt %	acid catalysis	adjusted to 7.2–7.4 with TRIS	chitosan	*Chlamydomonas reinhardtii* wildtype cc-124	photosynthetic activity and growth similar to free cells	continuous production of secondary metabolites (H_2_)	[52]
TEOS: 1.06 mol/L/22 wt %							[53]
Tetrakis(2-hydroxyethyl)orthosilicate: 2.20 mol/L/12.01 wt %	sol synthesis without additional acid or base	gelation at pH 6	--	*Porphyridium purpureum*	immobilization had a stabilizing effect, viability at elevated temperature; pigment fluorescence showed reusability and stability over 2 weeks	whole-cell biosensor for aqueous contaminants	[163]

**Table 2:** Overview of microalgae entrapment in aqueous silicates. APTMS: aminopropyl trimethoxysilane; ETES: ethyltriethoxysilane.

Silica Precursor and Concentration	Catalysis	pH Adjustment	Additives	Microalgae/ Cyanobacteria	Characteristics/ Viability	Purpose/Aim	Ref.
Sodium silicate: 0.16 mol/L/1.08 wt %	base catalysis	adjusted to 9 with HCl	--	*Dictyosphaerium chlorelloides*, *Scenedesmus intermedius*, *Scenedesmus* sp.	chlorophyll fluorescence stable for 3 weeks	whole-cell biosensor for aqueous contaminants	[129]
Sodium silicate + LUDOX^®^: 2.97 mol/L/14.86 wt %		adjusted to 7.5–8.0 with HCl		*Mesotaenium* sp., *Synechococcus* sp.	chlorophyll fluorescence; storage time 4 to 8 weeks		[164]
Sodium silicate + LUDOX^®^: 3.96 mol/L/18.34 wt %		adjusted to 7 with HCl	glycerol	*Chlorella vulgaris* CCAP 211/12	chlorophyll fluorescence, 4 weeks viable		[98]
					activity for 5 weeks		[124]
Sodium silicate + LUDOX^®^: 0.61 mol/L/3.04 wt % with APTMS: 0.63 mol/L/3.18 wt % with ETES: 0.64 mol/L/ 3.19 wt %		adjusted to 6 with HCl	--	*Anabaena flos-aqua*, *Chlorella vulgaris*, *Euglena gracilis*	organosilanes enable stable sensitivity to herbicides and metal ions; no investigation of the cells		[165]
Sodium silicate + LUDOX^®^: 0.37–2.93 mol/L/ 1.84–14.70 wt %		adjusted to 5–7 with HCl			chlorophyll fluorescence; “best gel” species-specific	biosensors and biotechnological application	[130]
Sodium silicate + LUDOX^®^: 5.9 mol/L/27.57 wt %		adjusted to 8 with HCl	glycerol	*Synechococcus* sp. PCC 6301, PCC 7002, *Cyanothece* PCC 7418	viability of cells over 3 months; bioactivity of cells	enabling (prolonged) viability and activity for important biotechnological applications, such as biofuels and (secondary) metabolites	[97]
Sodium silicate + LUDOX^®^: 3.7 mol/L/8.56 wt %				*Synechococcus* PCC 6301, PCC 7002, PCC 7418	chlorophyll intact for several months		[41]
Sodium silicate + LUDOX^®^: 5.9 mol/L/27.57 wt % Sodium silicate: 4.1 mol/L/19 wt %		adjusted to 7–8 with HCl	--	*Cyanidium caldarium* SAG 16.91	proliferation limited; photosynthesis in gels without additives; chlorophyll stable for 4 months		[42]

**Table 3:** Overview of microalgae entrapment in aqueous silicates with metal ion removal.

Silica Precursor and Concentration	Catalysis	pH Adjustment	Additives	Microalgae/ Cyanobacteria	Characteristics/ Viability	Purpose/Aim	Ref.
Sodium silicate: 4.7 mol/L/21.76 wt %; sodium silicate + SiO_2_ nanopowder: 5.13 mol/L/25.7 wt %	acid catalysis	adjusted to 6 with KOH	--	*Cyanidium caldarium* SAG 16.91	oxygen production for 75 days	CO_2_ mitigation, oxygenation of environments, production of secondary metabolites	[44]
				*Chlorella vulgaris* SAG 211–11b, *Botryococcus braunii* SAG 30.81	viable cells, chlorophyll fluorescence, oxygen production, proliferation limited		[43]
Sodium silicate: 0.55 mol/L/4.80 wt % sodium silicate + LUDOX^®^: 1.02–2.15 mol/L/9.41–23.24 wt %	without LUDOX^®^: acid catalysis with LUDOX^®^: base catalysis	adjusted to 7–8 with KOH (without LUDOX^®^) or HCl (with LUDOX^®^)	glycerol	*Synechococcus* sp. PCC 6301 and PCC 7002	preservation of the photosynthetic pigment of up to 35 weeks; oxygen production for 17 weeks	enabling (prolonged) viability and activity for important biotechnological applications, such as biofuels and (secondary) metabolites	[94]
Sodium silicate: 0.3–0.88 mol/L/7–25 wt %	acid catalysis	adjusted to 7.2–7.4 with TRIS	chitosan	*Chlamydomonas reinhardtii* wildtype cc-124	photosynthetic activity and growth similar to free cells	continuous production of secondary metabolites (H_2_)	[52]
sodium silicate: 0.88 mol/L/20 wt %							[53]

**Table 4:** Overview of microalgae entrapment in aminosilane-based silica hydrogels.

Silica Precursor and Concentration	Catalysis	Reduction of By-Product Concentration	pH Adjustment	Additives	Microalgae/ Cyanobacteria	Characteristics/ Viability	Purpose/Aim	Ref.
Tetra(*n*-propylamino)silane: 0.96 mol/L/25 wt %	base catalysis	--	adjusted to 7 with an unspecified acid	--	*Chlamydomonas reinhardtii* wild-type cc-124	photosynthetic activity drastically reduced over 2 h	entrapment of sensitive material in highly transparent hydrogels	[70]
Tetra(*n*-propylamino)silane: 0.19–0.96 mol/L/5–25 wt %	acid catalysis	removal of propylamine via ion exchanger	adjusted to 7.2–7.4 with TRIS buffer	chitosan	*Chlamydomonas reinhardtii* wild-type cc-124	photosynthetic activity and growth of entrapped micro-algae similar to free cells	continuous production of secondary metabolites (H_2_)	[52]
tetra(*n*-propylamino)silane: 0.96 mol/L/25 wt %								[53]

**Table 5:** Overview of core-shell and two-step entrapment of microalgae. TEOS: tetraethyl orthosilicate.

Silica Precursor and Concentration	Catalysis	Reduction of By-Product Concentration	pH Adjustment	Method	Microalgae/ Cyanobacteria	Characteristics/ Viability	Purpose/Aim	Ref.
Sodium siliate: 0.72 mol/L/ 37.56 wt %	acid catalysis	removal of the sodium ions via ion exchanger	pH adjusted to 5.1 with NaOH	hybrid core-shell beads	*Dunaliella tertiolecta*	oxygen production and chlorophyll fluorescence show photosynthetic activity for 13 months	enabling (prolonged) viability and activity for important biotechnological applications, like biofuels and (secondary) metabolites	[96]
								[132]
					*Chlamydomonas reinhardtii*	viability and cellular functionality for more than 4 months		[133]
TEOS: 1.66 mol/L/ 10.15 wt%	acid catalysis	evaporation of alcohols	adjusted with phosphate buffer pH 7	two-step entrapment	*Chlorella vulgaris*	chlorophyll preservation (green intensity) at UV irridation	development of robust silica hydrogels with CeO_2_ nano- particles that protects encapsulated cells for green energy	[126]
			adjusted with KOH		*Chlorella vulgaris*, *Pseudokirchneriella subcapitata*, *Chlamydomonas reinhardtii*	cell growth was unaffected by encapsulation	whole-cell biosensor for aqueous contaminants	[127]
Sodium silicate + LUDOX^®^: 7.95 mol/L/ 36.75 wt %	base catalysis	--	adjusted to 6.5 with HCl			chlorophyll fluorescence; growth in calcium alginate voids was hardly affected		[125]
						chlorophyll fluorescence		[128]
Sodium silicate + LUDOX^®^: 0.65–2.17 mol/L/3.53–11.04 wt %					*Chlorella vulgaris*, *Pseudokirchneriella subcapitata*,	investigation of silica concentration, ratio of precursors, thickness, and cell loading on sensor’s performance		[170]
TEOS + diamino-functionalized silane: 0.17 mol/L/3.16 wt %	acid catalysis	evaporation of alcohols	adjusted to 7.5 with HCl		*Chlorella vulgaris*	activity maintained for 8 weeks; cell growth in alginate voids observed		[131]

## Data Availability

Not applicable.
